# Thermal transport of Josephson junction based on two-dimensional electron gas

**DOI:** 10.1038/s41598-019-38704-6

**Published:** 2019-02-18

**Authors:** Xiaoxuan Luo, Yufeng Peng, Hongzhi Shen, Xuexi Yi

**Affiliations:** 1 Center for Quantum Sciences, Northeast Normal University, Changchun, 130117 China; 2 Center for Advanced Optoelectronic Functional Materials Research, and Key Laboratory for UV Light-Emitting Materials and Technology of Ministry of Education, Northeast Normal University, Changchun, 130024 China

## Abstract

We study the phase-dependent thermal transport of a short temperature-biased Josephson junction based on two-dimensional electron gas (2DEG) with both Rashba and Dresselhaus couplings. Except for thermal equilibrium temperature *T*, characters of thermal transport can also be manipulated by interaction parameter *h*_0_ and the parameter $|\frac{{{\boldsymbol{\lambda }}}_{{\boldsymbol{R}}}}{{{\boldsymbol{\beta }}}_{{\boldsymbol{R}}}}-\frac{{{\boldsymbol{\lambda }}}_{{\boldsymbol{L}}}}{{\beta }_{{\boldsymbol{L}}}}|$ . A larger value and a sharper switching behavior of thermal conductance can be obtained if *h*_0_ takes suitable values and $|\frac{{{\boldsymbol{\lambda }}}_{{\boldsymbol{R}}}}{{{\boldsymbol{\beta }}}_{{\boldsymbol{R}}}}-\frac{{{\boldsymbol{\lambda }}}_{{\boldsymbol{L}}}}{{{\boldsymbol{\beta }}}_{{\boldsymbol{L}}}}|$ is larger. Finally, we propose a possible experimental setup based on the discussed Josephson junction and find that the temperature of the right superconducting electrode *T*_*R*_ is influenced by the same three parameters in a similar way with thermal conductance. This setup may provide a valid method to select moderately-doped 2DEG materials and superconducting electrodes to control the change of temperature and obtain an efficient temperature regulator.

## Introduction

When it comes to modern computers, we have to talk about the disposal of excessive heat. In the past few decades, the level of integration of computer chips has been dramatically raised, the computational power becomes stronger but at the same time the excessive Joule heat production becomes one of the biggest problems deleterious to computational efficiency. As a consequence, we need to adopt valid methods to handle excessive heat and cool circuits.

This makes us consider about logical circuits based on thermal currents rather than traditional electrical conduction. At the same time, we have known that the thermal currents carried by thermal quasiparticles flowing through a temperature-biased Josephson junction connecting two superconducting electrodes will be a static-periodic function of phase difference between the electrodes^1,2^. That is to say, the variation of thermal currents is determined by the difference in phases of the two electrodes^3^. Based on this fact, people have been trying to open up a new area where they offer insight into the phase-dependent thermal transport in many hybrid superconducting structures or nanostructures^4^, with particular emphasis on overcoming the problems associated with excessive heat production and proposing high-efficiency logical devices for information transport. There are many achievements in this area, such as a Josephson heat interferometer^2^, the phase-dependent heat transport in submicron superconducting structures studied independently by two groups Petrashov *et al*. and Kastalskii *et al*.^4^, the manipulation of thermal transport in phononic devices^5^, the phase-coherent thermal transport through Josephson weak links^6^ and Josephson point contacts^7^, a quantum phase-dependent superconducting thermal-flux modulator^8^ based on superconducting quantum interference device (SQUID) and the treating process of quantum information^9,10^. Moreover, the magnetic-flux manipulation of phase-dependent heat transport by a double-loop thermal modulator^11,12^ and the phase control of heat transport by a first balanced Josephson thermal modulator^13^ become possible. In addition, a V-based nanorefrigerator^14^ and an electron refrigerator based on tunneling junctions^15^ were proposed to cool electrons, a radiation comb generator based on Josephson junction and the way of microwave Josephson quantum refrigeration based on the structure of SQUID were proposed by P. Solinas *et al*. in recent years, they found that the generator and the dynamics of phase of the refrigerator can be both controlled by external applied magnetic field due to Josephson effect^16–19^.

Lately, there exists an increasingly interest in topological Josephson junction and someone has put forward an efficient thermal switch based on two-dimensional S-TI-S Josephson junction laying on x-y plane [S denotes superconductor and TI represents topological insulator]^20^. This device can be adjusted by magnetic field perpendicular to x-y plane to reach a large relative temperature variation of 40%. Meanwhile its sharp switching behavior can be realized by a short length *L* and a low temperature *T* of the junction.

**Figure 1:**
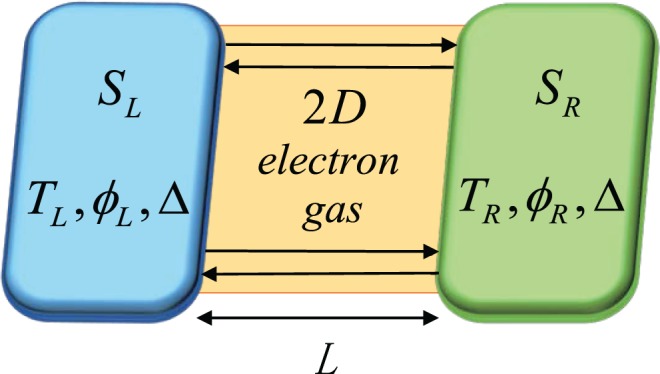
Sketch of a temperature-biased Josephson junction based on two-dimensional electron gas linked with two superconducting electrodes *S*_*L*,*R*_ on both sides.

Inspired by topological Josephson junction, in this paper we study a phase-coherent temperature-biased Josephson junction with a hybrid structure consisting of 2DEG (not TI) [see Fig. 1]. Although there have been so many research works towards 2DEG^4,21–24^, we wish to see the influence of material parameters on the thermal transport characters of the junction and try to find if there is another way to control thermal transport rather than the length of junction *L*. We demonstrate that the thermal transport (or thermal conductance) is affected by the thermal equilibrium temperature *T*, interaction parameter *h*_0_ deriving from concentration of magnetic impurities describing the interaction between impurities and quasiparticles and the parameter $|\frac{{\lambda }_{R}}{{\beta }_{R}}-\frac{{\lambda }_{L}}{{\beta }_{L}}|$ (*λ*_*L*,*R*_, *β*_*L*,*R*_ represents Rashba and Dresselhaus coupling respectively with subscripts *L*, *R* representing the left and right superconducting electrode [see Fig. 1].) Moreover, we propose a possible experimental setup based on the Josephson junction discussed above and find that the temperature of right-hand superconductor *T*_*R*_ exhibits similar behavior with thermal conductance under the impact of the same parameters. Compared with our model, S-TI-S Josephson junction does not possess the above-mentioned three factors affecting thermal transport, therefore our model has obvious advantages.

This paper is organized as follow. Firstly, we introduce a theoretical model of S-2DEG-S Josephson junction described by Bogoliubov-de Gennes Hamiltonian and deduce the transmission probability functions and thermal conductance of the junction. Then, we analyze the results. Moreover we propose an experimental realization to study the characters of *T*_*R*_ with practical parameters. Finally, we do some discussion and draw conclusions.

## Methods

### System and Bogoliubov-de Gennes Hamiltonian

The basic model we analyze here is a Josephson junction based on 2DEG in the x-y plane showed in Fig. 1, but for simplicity we will consider about a short junction with *L* = 0 in the following text. Superconducting electrode on the left or right side is named as *S*_*L*,*R*_ with superconducting phase *ϕ*_*L*,*R*_ and temperature *T*_*L*,*R*_ respectively. Considering about *T*_*L*_ > *T*_*R*_, there exists a temperature bias Δ*T* = *T*_*L*_ − *T*_*R*_, therefore thermal currents in linear response flowing through the junction can be expressed as *J* = *κ*(*ϕ*)Δ*T*, prior to which we need to consider about thermal conductance *κ*(*ϕ*) with the phase difference *ϕ* = *ϕ*_*R*_ − *ϕ*_*L*_.

**Figure 2:**
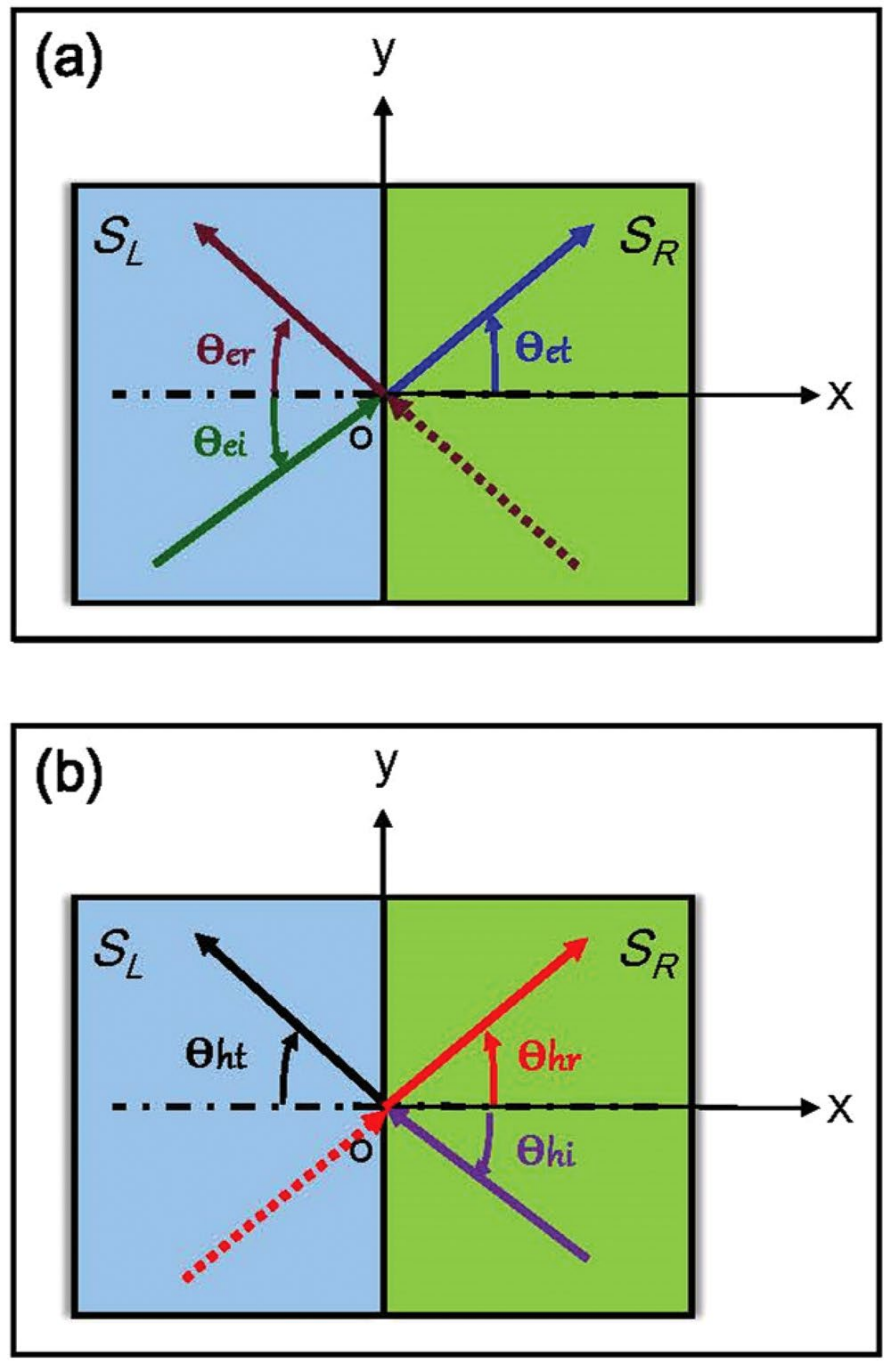
Movement of quasiparticles at the boundary. *θ*_*ei*,*hi*_, *θ*_*er*,*hr*_ and *θ*_*et*,*ht*_ are angles of incidence, reflection and transmission for electron- or holelike quasiparticles. (**a**) Movement of electron-like quasiparticles at the boundary, solid lines represent right-moving quasiparticles $({\theta }_{ei}\in [0,\frac{\pi }{2}])$ and dashed lines denotes opposite-moving quasiparticles $(\pi -{\theta }_{ei}\in [\frac{\pi }{2},\pi ])$. (**b**) Movement of hole-like quasiparticles at the boundary, solid lines represent left-moving quasiparticles $({\theta }_{hi}\in [0,\frac{\pi }{2}])$ and dashed lines denotes opposite-moving quasiparticles $(\pi -{\theta }_{hi}\in [\frac{\pi }{2},\pi ])$.

First of all we pay attention to Bogoliubov-de Gennes Hamiltonian describing quasiparticles in Josephson junction^25,26^

1 $${\hat{H}}_{BdG}=(\begin{array}{cc}{\hat{h}}_{\overrightarrow{k}} & i{\hat{\sigma }}_{y}{\rm{\Delta }}{e}^{i{\varphi }_{r}}\\ -i{\hat{\sigma }}_{y}{\rm{\Delta }}{e}^{-i{\varphi }_{r}} & -{\hat{h}}_{-\overrightarrow{k}}^{\ast }\end{array}),$$

which acts on the energy eigenstates of electron- and holelike quasiparticles. Δ is superconducting energy gap only existing in superconductor electrodes [see Fig. 1]. ${\rm{\Delta }}{e}^{i{\varphi }_{r}}$ denotes superconducting order parameter with *r* = *L*, *R*. The specific form of the element ${\hat{h}}_{\overrightarrow{k}}$ on main diagonal in Eq. (1) is written as^27–29^

2 $${\hat{h}}_{\overrightarrow{k}}={d}_{x}{\hat{\sigma }}_{x}+{d}_{y}{\hat{\sigma }}_{y}+{d}_{z}{\hat{\sigma }}_{z}-\mu {\hat{\sigma }}_{0},$$

where *d*_*x*_ = ℏ(*λ*_*r*_*k*_*y*_ − *β*_*r*_*k*_*x*_), *d*_*y*_ = ℏ(−*λ*_*r*_*k*_*x*_ + *β*_*r*_*k*_*y*_) and *d*_*z*_ = *h*_0_. *h*_0_ is the material interaction parameter^30^ taking a value from $[0,\,\,+\,{\rm{\infty }})$ in principle, due to the fact that there is no restrictions on interaction strength between quasiparticles and impurities, *μ* the chemical potential, *λ*_*r*_ and *β*_*r*_ the spin-orbit coupling parameters with *r* = *L*, *R*^31^ (In this paper, we consider *λ*_*L*_ *≠* *λ*_*R*_, *β*_*L*_ *≠* *β*_*R*_ in general). ${\hat{\sigma }}_{x}$, ${\hat{\sigma }}_{y}$ and ${\hat{\sigma }}_{z}$ are Pauli matrices with ${\hat{\sigma }}_{0}$ the unit matrix. Wave vector of quasiparticles is written as $\overrightarrow{k}=({k}_{x},{k}_{y})=k(\cos \,{\theta }_{i},\,\sin \,{\theta }_{i})$, *θ*_*i*_ is incident angle of quasiparticles taking *θ*_*ei*_ or *θ*_*hi*_ [see Fig. 2] and *k* is the module of wave vector (the specific form of *k* is calculated in the following).

In order to simplify calculation, we firstly write ${\hat{h}}_{\overrightarrow{k}}$ in Eq. (2) as the following matrix form

3 $${\hat{h}}_{\overrightarrow{k}}=(\begin{array}{cc}-\mu +{h}_{0} & {d}_{x}-i{d}_{y}\\ {d}_{x}+i{d}_{y} & -\mu -{h}_{0}\end{array}).$$

Then plugging *d*_*x*_ = ℏ(*λ*_*r*_*k*_*y*_ − *β*_*r*_*k*_*x*_), *d*_*y*_ = ℏ(−*λ*_*r*_*k*_*x*_ + *β*_*r*_*k*_*y*_) and *k*_*x*_ = *k*cos*θ*_*i*_, *k*_*y*_ = *k*sin*θ*_*i*_ into Eq. (3), we have

4 $${\hat{h}}_{\overrightarrow{k}}=(\begin{array}{cc}-\mu +{h}_{0} & \hslash k(i{\lambda }_{r}{e}^{-i{\theta }_{i}}-{\beta }_{r}{e}^{i{\theta }_{i}})\\ \hslash k(-i{\lambda }_{r}{e}^{i{\theta }_{i}}-{\beta }_{r}{e}^{-i{\theta }_{i}}) & -\mu -{h}_{0}\end{array}).$$

$-{\hat{h}}_{-\overrightarrow{k}}^{\ast }$ can be calculated by the same method. At last, plugging the matrix form of the Bogoliubov-de Gennes Hamiltonian ${\hat{H}}_{BdG}$ into an eigenequation describing the Josephson junction

5 $${\hat{H}}_{BdG}{\rm{\Psi }}(\overrightarrow{r})=\omega {\rm{\Psi }}(\overrightarrow{r}),$$

where *ω* is energy eigenvalue of quasiparticles and we take it as given quantity in the following.

### Eigenfunctions of Quasiparticles

Through solving

6 $$|{\hat{H}}_{BdG}-\omega |=0,$$

we get the above-mentioned specific form of *k* for electron- and hole-like quasiparticles as

7 $${k}_{e}=\sqrt{\frac{{\mu }^{2}+{\omega }^{2}-{{\rm{\Delta }}}^{2}-{h}_{0}^{2}+2\sqrt{{{\rm{\Delta }}}^{2}{h}_{0}^{2}-{{\rm{\Delta }}}^{2}{\mu }^{2}+{\mu }^{2}{\omega }^{2}}}{{\hslash }^{2}({\lambda }_{r}^{2}+{\beta }_{r}^{2}-2{\lambda }_{r}{\beta }_{r}\,\sin \,2{\theta }_{ei})}}$$

and

8 $${k}_{h}=\sqrt{\frac{{\mu }^{2}+{\omega }^{2}-{{\rm{\Delta }}}^{2}-{h}_{0}^{2}-2\sqrt{{{\rm{\Delta }}}^{2}{h}_{0}^{2}-{{\rm{\Delta }}}^{2}{\mu }^{2}+{\mu }^{2}{\omega }^{2}}}{{\hslash }^{2}({\lambda }_{r}^{2}+{\beta }_{r}^{2}-2{\lambda }_{r}{\beta }_{r}\,\sin \,2{\theta }_{hi})}}$$

containing *θ*_*ei*,*hi*_ and *λ*_*r*_, *β*_*r*_ (These two kinds of spin-orbit coupling critical for thermal transport can not be zero at the same time when calculating with this method). To avoid confusion with the reverse movement of quasiparticles, we mark *k*_*e*,*h*_ as ${k}_{{e}_{1r},{h}_{1r}}$ respectively. Then we take *k* as ${k}_{{e}_{1r}}$ when solving eigenequation Eq. (5) and obtain the eigenfunction of right-moving electron-like quasiparticles with *θ*_*ei*_

9 $${{\rm{\Psi }}}_{1}(\overrightarrow{r})=(\begin{array}{c}\frac{2{\rm{\Delta }}(\mu +{h}_{0})\hslash {k}_{{e}_{1r}}(i{\lambda }_{r}{e}^{-i{\theta }_{ei}}-{\beta }_{r}{e}^{i{\theta }_{ei}})}{{A}_{e}^{1/2}}\\ -\frac{{\rm{\Delta }}[2(\mu +{h}_{0})(\,-\,\mu +{h}_{0}-\omega )+{\chi }_{e}]}{{A}_{e}^{1/2}}\\ -\frac{2{{\rm{\Delta }}}^{2}(\mu +{h}_{0})+(\mu +{h}_{0}-\omega ){\chi }_{e}}{{A}_{e}^{1/2}}{e}^{-i{\varphi }_{r}}\\ \frac{{\chi }_{e}\hslash {k}_{{e}_{1r}}(i{\lambda }_{r}{e}^{-i{\theta }_{ei}}-{\beta }_{r}{e}^{i{\theta }_{ei}})}{{A}_{e}^{1/2}}{e}^{-i{\varphi }_{r}}\end{array}){e}^{i{\overrightarrow{k}}_{{e}_{1r}}\overrightarrow{r}}.$$

Similarly, we take *k* as ${k}_{{h}_{1r}}$ and obtain the eigenfunction of left-moving hole-like quasiparticles incident with *θ*_*hi*_

10 $${{\rm{\Psi }}}_{2}(\overrightarrow{r})=(\begin{array}{c}\frac{2{\rm{\Delta }}(\mu +{h}_{0})\hslash {k}_{{h}_{1r}}(i{\lambda }_{r}{e}^{-i{\theta }_{hi}}-{\beta }_{r}{e}^{i{\theta }_{hi}})}{{A}_{h}^{1/2}}\\ -\frac{{\rm{\Delta }}[2(\mu +{h}_{0})(\,-\,\mu +{h}_{0}-\omega )+{\chi }_{h}]}{{A}_{h}^{1/2}}\\ -\frac{2{{\rm{\Delta }}}^{2}(\mu +{h}_{0})+(\mu +{h}_{0}-\omega ){\chi }_{h}}{{A}_{h}^{1/2}}{e}^{-i{\varphi }_{r}}\\ \frac{{\chi }_{h}\hslash {k}_{{h}_{1r}}(i{\lambda }_{r}{e}^{-i{\theta }_{hi}}-{\beta }_{r}{e}^{i{\theta }_{hi}})}{{A}_{h}^{1/2}}{e}^{-i{\varphi }_{r}}\end{array}){e}^{i{\overrightarrow{k}}_{{h}_{1r}}\overrightarrow{r}}.$$

The eigenfunctions ${{\rm{\Psi }}}_{3,4}(\overrightarrow{r})$ of opposite-moving quasiparticles can be obtained from ${{\rm{\Psi }}}_{1,2}(\overrightarrow{r})$ through substituting the incident angle *θ*_*ei*,*hi*_ by *π* − *θ*_*ei*,*hi*_ respectively. Therefore the eigenfunction ${{\rm{\Psi }}}_{3}\,(\overrightarrow{r})$ of left-moving electron-like quasiparticles is

11 $${{\rm{\Psi }}}_{3}(\overrightarrow{r})=(\begin{array}{c}\frac{2{\rm{\Delta }}(\mu +{h}_{0})\hslash {k}_{{e}_{2r}}(\,\,-\,\,i{\lambda }_{r}{e}^{i{\theta }_{ei}}+{\beta }_{r}{e}^{-i{\theta }_{ei}})}{{A}_{e}^{1/2}}\\ -\frac{{\rm{\Delta }}[2(\mu +{h}_{0})(\,\,-\,\,\mu +{h}_{0}-\omega )+{\chi }_{e}]}{{A}_{e}^{1/2}}\\ -\frac{2{{\rm{\Delta }}}^{2}(\mu +{h}_{0})+(\mu +{h}_{0}-\omega ){\chi }_{e}}{{A}_{e}^{1/2}}{e}^{-i{\varphi }_{r}}\\ \frac{{\chi }_{e}\hslash {k}_{{e}_{2r}}(\,\,-\,\,i{\lambda }_{r}{e}^{i{\theta }_{ei}}+{\beta }_{r}{e}^{-i{\theta }_{ei}})}{{A}_{e}^{1/2}}{e}^{-i{\varphi }_{r}}\end{array}){e}^{i{\overrightarrow{k}}_{{e}_{2r}}\overrightarrow{r}},$$

the eigenfunction ${{\rm{\Psi }}}_{4}(\overrightarrow{r})$ of right-moving hole-like quasiparticles is

12 $${{\rm{\Psi }}}_{4}(\overrightarrow{r})=(\begin{array}{c}\frac{2{\rm{\Delta }}(\mu +{h}_{0})\hslash {k}_{{h}_{2r}}(-i{\lambda }_{r}{e}^{i{\theta }_{hi}}+{\beta }_{r}{e}^{-i{\theta }_{hi}})}{{A}_{h}^{1/2}}\\ -\frac{{\rm{\Delta }}[2(\mu +{h}_{0})(-\mu +{h}_{0}-\omega )+{\chi }_{h}]}{{A}_{h}^{1/2}}\\ -\frac{2{{\rm{\Delta }}}^{2}(\mu +{h}_{0})+(\mu +{h}_{0}-\omega ){\chi }_{h}}{{A}_{h}^{1/2}}{e}^{-i{\phi }_{r}}\\ \frac{{\chi }_{h}\hslash {k}_{{h}_{2r}}(-i{\lambda }_{r}{e}^{i{\theta }_{hi}}+{\beta }_{r}{e}^{-i{\theta }_{hi}})}{{A}_{h}^{1/2}}{e}^{-i{\phi }_{r}}\end{array}){e}^{i{\overrightarrow{k}}_{{h}_{2r}}\overrightarrow{r}}.$$

The specific expressions of parameters ${k}_{{e}_{1r},{e}_{2r}},{k}_{{h}_{1r},{h}_{2r}},{\chi }_{e,h},{A}_{e,h},{\overrightarrow{k}}_{{e}_{1r},{e}_{2r}},{\overrightarrow{k}}_{{h}_{1r},{h}_{2r}}$ and $\overrightarrow{r}$ in Eqs (9–12) can be found in Supplementary Note of Supplementary Information online.

### Transmission Probability and Thermal Conductance

Now we consider the situation of a right-moving electron-like quasiparticle emitting from the left-hand superconductor. In the process of incidence, this quasiparticle stimulates three other kinds of quasiparticles so the wave functions of the two superconducting regions can be written as

13 $${{\rm{\Psi }}}_{L}(\overrightarrow{r})={{\rm{\Psi }}}_{1}(\overrightarrow{r})+{r}_{e}{{\rm{\Psi }}}_{3}(\overrightarrow{r})+{r}_{h}{{\rm{\Psi }}}_{2}(\overrightarrow{r}),$$

14 $${{\rm{\Psi }}}_{R}(\overrightarrow{r})={t}_{e}{{\rm{\Psi }}}_{1}(\overrightarrow{r})+{t}_{h}{{\rm{\Psi }}}_{4}(\overrightarrow{r}).$$

For the sake of convenience in the calculation, ${{\rm{\Psi }}}_{L,R}(\overrightarrow{r})$ are rewritten as

15 $${{\rm{\Psi }}}_{L}(\overrightarrow{r})={{\rm{\Psi }}}_{1L}(\overrightarrow{r})+{r}_{e}{{\rm{\Psi }}}_{3L}(\overrightarrow{r})+{r}_{h}{{\rm{\Psi }}}_{2L}(\overrightarrow{r}),$$

16 $${{\rm{\Psi }}}_{R}(\overrightarrow{r})={t}_{e}{{\rm{\Psi }}}_{1R}(\overrightarrow{r})+{t}_{h}{{\rm{\Psi }}}_{4R}(\overrightarrow{r}),$$

where ${{\rm{\Psi }}}_{1{\rm{L}},1{\rm{R}}}(\overrightarrow{r}),{{\rm{\Psi }}}_{2L}(\overrightarrow{r}),{{\rm{\Psi }}}_{3L}(\overrightarrow{r})$ and ${{\rm{\Psi }}}_{4R}(\overrightarrow{r})$ are obtained from Eqs (9–12) by replacing subscript *r* with *L*, *R*. We simply express ${{\rm{\Psi }}}_{1{\rm{L}},1{\rm{R}}}(\overrightarrow{r}),{{\rm{\Psi }}}_{2L}(\overrightarrow{r}),{{\rm{\Psi }}}_{3L}(\overrightarrow{r})$ and ${{\rm{\Psi }}}_{4R}(\overrightarrow{r})$ as

$${{\rm{\Psi }}}_{1{\rm{L}}}(\overrightarrow{r})={(\begin{array}{cccc}{a}_{1L} & {b}_{1L} & {c}_{1L} & {d}_{1L}\end{array})}^{{\rm{T}}}{e}^{i{\overrightarrow{k}}_{{e}_{1L}}\overrightarrow{r}},{{\rm{\Psi }}}_{1{\rm{R}}}(\overrightarrow{r})={(\begin{array}{cccc}{a}_{1R} & {b}_{1R} & {c}_{1R} & {d}_{1R}\end{array})}^{{\rm{T}}}{e}^{i{\overrightarrow{k}}_{{e}_{1R}}\overrightarrow{r}},$$

$${{\rm{\Psi }}}_{2{\rm{L}}}(\overrightarrow{r})={(\begin{array}{cccc}{a}_{2} & {b}_{2} & {c}_{2} & {d}_{2}\end{array})}^{{\rm{T}}}{e}^{i{\overrightarrow{k}}_{{h}_{1L}}\overrightarrow{r}},$$

$${{\rm{\Psi }}}_{3{\rm{L}}}(\overrightarrow{r})={(\begin{array}{cccc}{a}_{3} & {b}_{3} & {c}_{3} & {d}_{3}\end{array})}^{{\rm{T}}}{e}^{i{\overrightarrow{k}}_{{e}_{2L}}\overrightarrow{r}},$$

$${{\rm{\Psi }}}_{4{\rm{R}}}(\overrightarrow{r})={(\begin{array}{cccc}{a}_{4} & {b}_{4} & {c}_{4} & {d}_{4}\end{array})}^{{\rm{T}}}{e}^{i{\overrightarrow{k}}_{{h}_{2R}}\overrightarrow{r}}.$$

In the following text, we set the potential barrier at electron-gas–superconductor interface to be zero. Originated from the way of nonequilibrium quasiclassical Green functions^6,7,32,33^, we demand the continuity of the two wave functions in Eqs (15) and (16) at the interface (x = 0). Therefore the transmission coefficients *t*_*e*_, *t*_*h*_ can be obtained (see Supplementary Eqs S1 and S2 in Supplementary Information online). Thermal transmission possibility of electron- and hole-like quasiparticles are written as *T*_*e*,*h*_ = |*t*_*e*,*h*_|^2^. Then plugging specific expressions of ${k}_{{e}_{1r},{e}_{2r}},{k}_{{h}_{1r},{h}_{2r}}$ into *T*_*e*,*h*_, considering that *ω* > 0 and ${k}_{{e}_{1r}},{k}_{{h}_{1r}}$ are real numbers greater than zero, the thermal conductance can be determined by^26^

17 $$\kappa (\varphi )=\{\begin{array}{cc}{\int }_{-{h}_{0}+\sqrt{{\mu }^{2}+{{\rm{\Delta }}}^{2}}}^{+{\rm{\infty }}}{f}_{e}d\omega +{\int }_{{h}_{0}+\sqrt{{\mu }^{2}+{{\rm{\Delta }}}^{2}}}^{+{\rm{\infty }}}{f}_{h}d\omega , & {h}_{0}\in [0,\sqrt{{\mu }^{2}+{{\rm{\Delta }}}^{2}}]\\ {\int }_{{h}_{0}-\sqrt{{\mu }^{2}+{{\rm{\Delta }}}^{2}}}^{+{\rm{\infty }}}{f}_{e}d\omega +{\int }_{{h}_{0}+\sqrt{{\mu }^{2}+{{\rm{\Delta }}}^{2}}}^{+{\rm{\infty }}}{f}_{h}d\omega , & {h}_{0}\in (\sqrt{{\mu }^{2}+{{\rm{\Delta }}}^{2}},+{\rm{\infty }})\end{array},$$

where,

18 $${f}_{e}=\frac{{\omega }^{2}{T}_{e}}{4h{k}_{B}{T}^{2}\,\cos \,{{\rm{h}}}^{2}(\frac{\omega }{2{k}_{B}T})},{f}_{h}=\frac{{\omega }^{2}{T}_{h}}{4h{k}_{B}{T}^{2}\,\cos \,{h}^{2}(\frac{\omega }{2{k}_{B}T})}$$

and *k*_*B*_ is Boltzmann constant.

Let us analyze the characters of phase-dependent thermal conductance *κ*(*ϕ*) defined in Eq. (17) in unit of the thermal conductance quantum ${G}_{Q}={\pi }^{2}{k}_{B}^{2}T/(3h)$ when incident angles *θ*_*ei*,*hi*_, thermal equilibrium temperature *T*, interaction parameter *h*_0_ and spin-orbit coupling parameters *λ*_*r*_, *β*_*r*_ both change.

## Results

### Characters of Thermal Conductance-

In follow-up discussion, we make a few remarks. The first is *κ*(*ϕ*) is replaced with *κ*(*ϕ*)/*G*_*Q*_^20,26^. The second is we take *ϕ*_*L*_ = 0 and *ϕ*_*R*_ ∈ [0, 2*π*], thus phase difference *ϕ* = *ϕ*_*R*_, but thermal conductance is still expressed as *κ*(*ϕ*)/*G*_*Q*_ not *κ*(*ϕ*_*R*_)/*G*_*Q*_ and *ϕ* in abscissa of figures will be written as *ϕ*_*R*_ because *ϕ*_*R*_ is the variable actually. The third is we write *θ*_*ei*,*hi*_ as *θ*_*e*,*h*_ for convenience.

**Figure 3:**
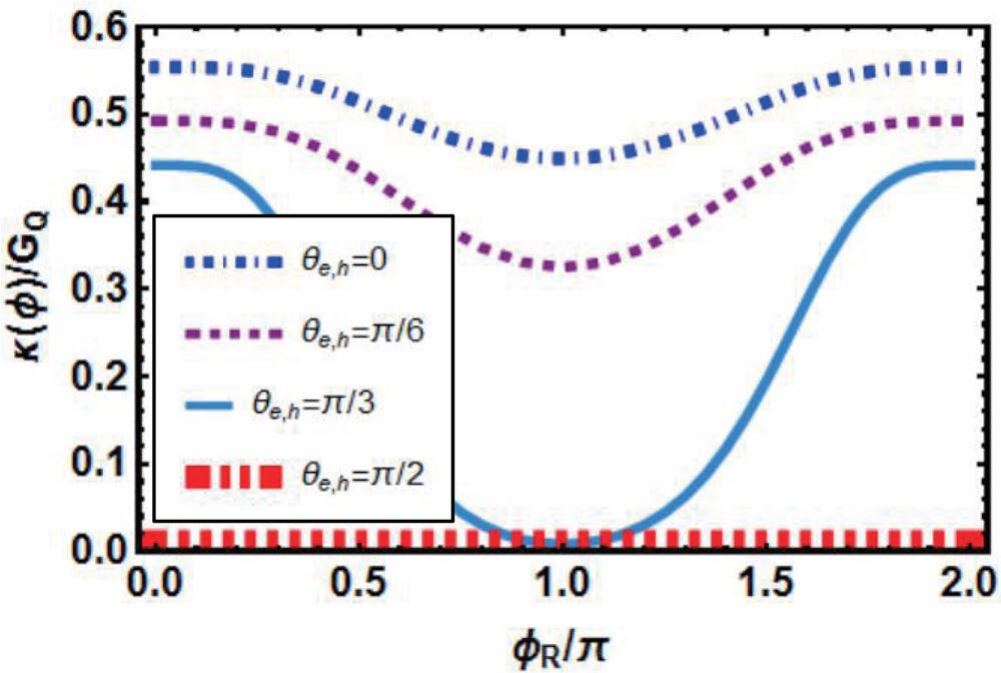
Phase-dependence of thermal conductance *κ*(*ϕ*)/*G*_*Q*_ with different incident angles. Here, *μ* = 1.38 *meV*, *h*_0_ = 1.38 *meV*, *T* = 100 *mK*, Δ = 0.15 *meV* and spin-orbit coupling parameters are *λ*_*L*_ = 23, *β*_*L*_ = 65, *λ*_*R*_ = 30, *β*_*R*_ = 16 with unit  *meVnm*/ℏ (Except for special instructions, the values of these four parameters are invariable in this paper).

Phase-dependence of thermal conductance *κ*(*ϕ*)/*G*_*Q*_ with change of the angle of incidence *θ*_*e*,*h*_ is shown in Fig. 3. To illustrate the characters of thermal transport simply and intuitively, four different incident angles belonging to $[0,\,\frac{\pi }{2}]$ are selected (Angles from $[-\frac{\pi }{2},\,0]$ are not considered here due to the symmetry of our model). Phase-dependence of thermal conductance are similar among different angles of incidence except ${\theta }_{e,h}=\frac{\pi }{2}$. More importantly, thermal conductance becomes smaller with the increase of *θ*_*e*,*h*_ when *ϕ*_*R*_ is constant. That is to say, thermal currents formed by normal-incident quasiparticles (*θ*_*e*,*h*_ = 0) make the greatest contribution to thermal transport along *x* direction, especially when *ϕ*_*R*_ = *π*. Incidence with large angles such as ${\theta }_{e,h}=\frac{\pi }{3},\frac{\pi }{2}$ makes little contribution basically, especially when ${\theta }_{e,h}=\frac{\pi }{2}$, thermal conductance becomes zero because quasiparticles move only in the *y* direction and do no contribution to thermal currents flowing in *x*-direction. In addition, the other parameters affecting thermal transport (such as *T*, *h*_0_ and *λ*_*r*_, *β*_*r*_) are independent of *θ*_*e*,*h*_. In consideration of the above analysis results, we will focus on normal incidence in the following discussion.

**Figure 4:**
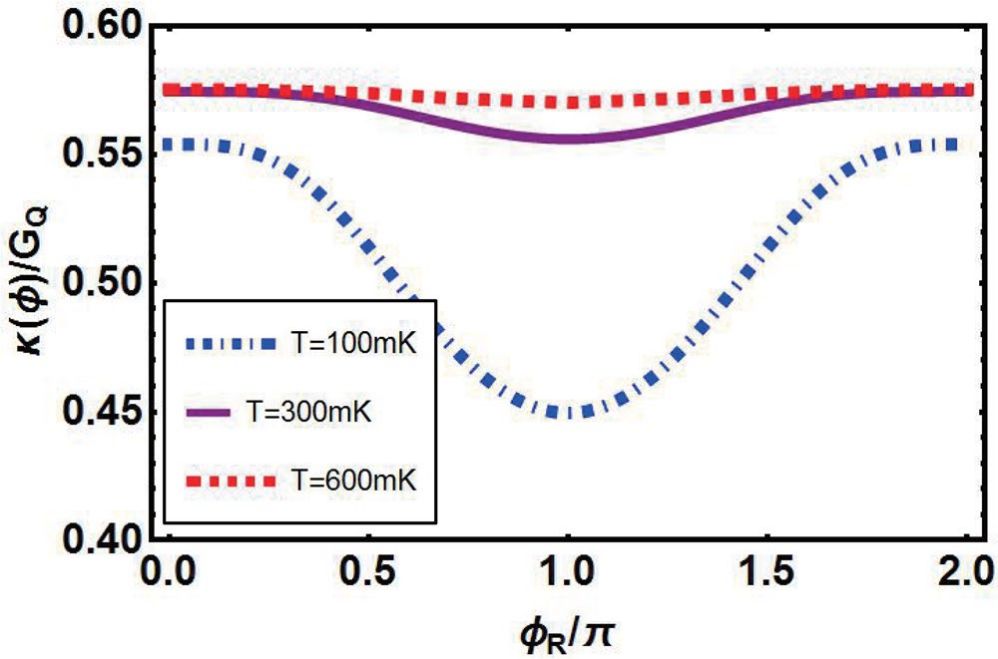
Phase-dependence of thermal conductance *κ*(*ϕ*)/*G*_*Q*_ at different temperatures *T* with *μ* = 1.38 *meV*, *h*_0_ = 1.38 *meV*, *T* = 100 *mK*, Δ = 0.15 *meV*.

First of all, we discuss about phase-dependence of *κ*(*ϕ*)/*G*_*Q*_ with different temperature *T* of heat reservoir^26^. Here *h*_0_ is taken as 1.38 *meV*^27,30^, see Fig. 4. There are three curves denoting situations with temperature *T* = 100 *mK*, 300 *mK* and 600 *mK* respectively. At low temperature *T* = 100 *mK*, thermal conductance is suppressed. This phenomenon is attributed to the fact that the amount of thermal excited quasiparticles with adequate energy to participate in thermal transport is too small to producing thermal currents strong enough when *T* is quite small. At the point of *ϕ*_*R*_ = *π*, the thermal conductance is suppressed. Upon enlarging *ϕ*_*R*_ from *π* to 2*π* or shrinking to 0, a great deal of thermal quasiparticles cross over the energy gap and thermal conductance climbs up with the variation of *ϕ*_*R*_ moderately. At last it reaches the maximum value when *ϕ*_*R*_ = 0, 2*π*. In this change process, thermal conductance varies from 0.45 up to about 0.55 and the relative variation equals to 22.2%. With the increase of temperature *T*, the value of thermal conductance at each point of the curve is raised, particularly the minimum value goes up to 0.51 for *T* = 300 *mK* and 0.54 for *T* = 600 *mK*. This is because more and more thermal quasiparticles are excited with the increase of *T*. As a result, the change of curves become increasingly moderate.

**Figure 5:**
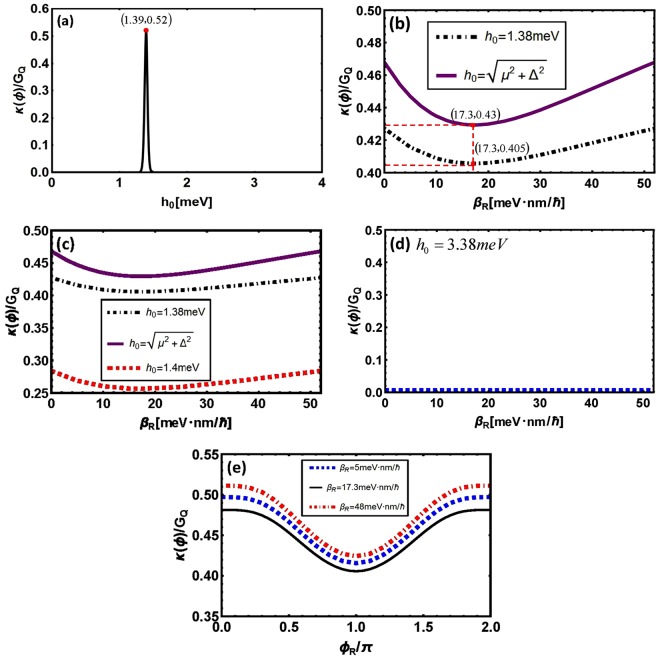
(**a**) *h*_0_-dependence of thermal conductance. (**b**–**d**) *β*_*R*_-dependence of thermal conductance with variable *h*_0_. (**e**) Phase-dependence of thermal conductance with variable *β*_*R*_ and *h*_0_ = 1.38 *meV*. (**b**–**e**) have the same parameters as *μ* = 1.38 *meV*, *T* = 100 *mK*, Δ = 0.15 *meV* and ${\lambda }_{L}=23\sqrt{3},{\beta }_{L}=23,{\lambda }_{R}=30$ with unit  *meVnm*/ℏ.

Secondly, we consider about another case where *h*_0_ becomes variable while *T* and *ϕ*_*R*_ both remain constant, see Fig. 5(a). As we know, the interaction parameter *h*_0_ describes the interaction between impurities and quasiparticles^30^, here we only consider *h*_0_ ∈ [0 *meV*, 4 *meV*] for convenience. Through analysis we observe that thermal conductance keeps at zero when *h*_0_ ∈ [0 *meV*, 1.35 *meV*], then it increases rapidly up to a “peak” located at about (1.39, 0.52), subsequently decreases to zero when *h*_0_ increases to about 1.43 *meV* and remains unchanged. Interestingly, the abscissa of the “peak” *h*_0_ = 1.39 *meV* coincides with the value $\sqrt{{\mu }^{2}+{{\rm{\Delta }}}^{2}}$ basically.

Thirdly, we plot out *β*_*R*_-dependence of *κ*(*ϕ*)/*G*_*Q*_ with variable *h*_0_ as shown in Fig. 5(b–d). Through analysis we find that the two curves in Fig. 5(b) with different *h*_0_ have one thing in common, that is when *β*_*R*_ ≈ 17.3 *meVnm*/ℏ thermal conductance will reach a smaller value, then the curves will go up obviously with the change of *β*_*R*_. Defining a parameter $|\frac{{\lambda }_{R}}{{\beta }_{R}}-\frac{{\lambda }_{L}}{{\beta }_{L}}|$ representing the difference of relative intensity of two different spin-orbit couplings, then it can be observed that $|\frac{{\lambda }_{R}}{{\beta }_{R}}-\frac{{\lambda }_{L}}{{\beta }_{L}}|=0$ corresponds to the smaller value of thermal conductance (Basically, it can be called the minimum value.). When *β*_*R*_ gradually moves away from the point 17.3 *meVnm*/ℏ, $|\frac{{\lambda }_{R}}{{\beta }_{R}}-\frac{{\lambda }_{L}}{{\beta }_{L}}|$ becomes larger and thermal conductance increases according to the curves in Fig. 5(b).

Another thing worth noting is that we just plot two curves in Fig. 5(b) as an example, actually the above-mentioned feature will appear when values of *h*_0_ are taken around $\sqrt{{\mu }^{2}+{{\rm{\Delta }}}^{2}}$, see Fig. 5(c). However it will disappear when *h*_0_ is far away from $\sqrt{{\mu }^{2}+{{\rm{\Delta }}}^{2}}$, see Fig. 5(d). Combined with Fig. 5(a), we observe that a larger value and a sharper switching behavior of thermal conductance will be obtained when the impurity concentration of 2DEG makes *h*_0_ equal to $\sqrt{{\mu }^{2}+{{\rm{\Delta }}}^{2}}$ or around it.

Moreover, the influence of *β*_*R*_ on the phase-dependence of thermal transport is plotted in Fig. 5(e), where the curve corresponding to *β*_*R*_ = 17.3 *meVnm*/ℏ (or ${\beta }_{R}=\frac{30}{\sqrt{3}}meVnm/\hslash$) is at the bottom. Whether *β*_*R*_ gets smaller or larger, the curves will move up, at the same time the change of curves accelerates. If we use the parameter $|\frac{{\lambda }_{R}}{{\beta }_{R}}-\frac{{\lambda }_{L}}{{\beta }_{L}}|$, the above analysis shows that the a larger value and a sharper switching behavior of thermal conductance will be obtained by a larger $|\frac{{\lambda }_{R}}{{\beta }_{R}}-\frac{{\lambda }_{L}}{{\beta }_{L}}|$.

**Figure 6:**
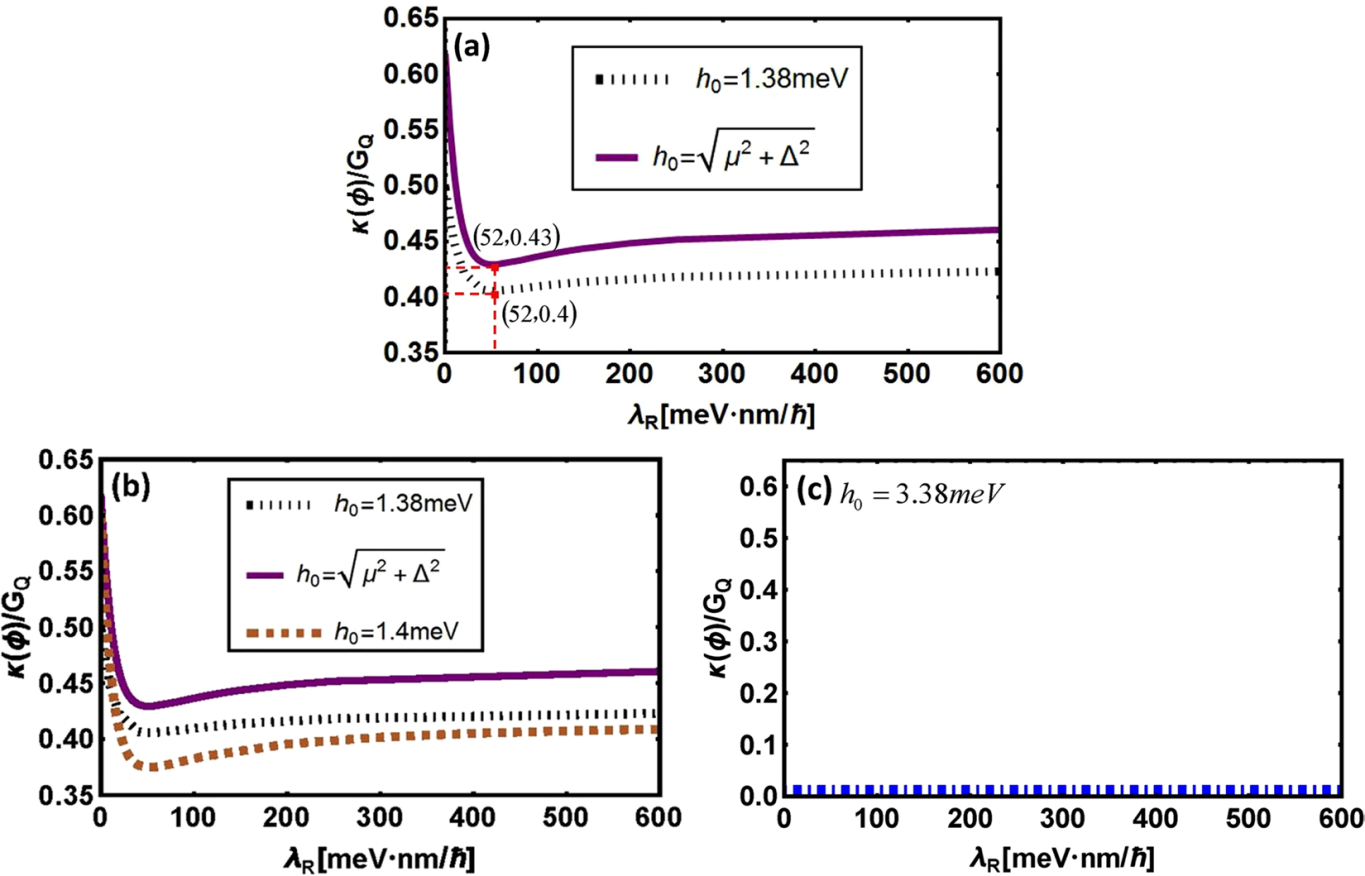
*λ*_*R*_-dependence of thermal conductance with variable *h*_0_. Here, *μ* = 1.38 *meV*, *T* = 100 *mK*, Δ = 0.15 *meV* and *ϕ*_*R*_ = *π*. Spin-orbit coupling parameters are taken as ${\lambda }_{L}=23\sqrt{3},{\beta }_{L}=23,\,{\beta }_{R}=30$ with unit *meVnm*/ℏ.

Finally, *λ*_*R*_-dependence of *κ*(*ϕ*)/*G*_*Q*_ with variable *h*_0_ are plotted in Fig. 6(a–c). Similar with Fig. 5(b–c) and Fig. 5(d), the same results will be obtained as above mentioned. In addition, *λ*_*R*_ has similar effect on the phase-dependence of thermal transport as *β*_*R*_ does because the change of thermal conductance under the impact of *β*_*R*_ and *λ*_*R*_ are similar.

## Possible Application

Knowing the characters of thermal conductance theoretically, in the following we put forward a possible experimental setup to realize a temperature regulator.

Here the setup is based on the Josephson junction discussed above and there is an additional normal metal contactor served as heater to inject heat into the left superconductor and a N-I-S junction performing as thermometry^2^ to measure the temperature of the right superconductor in stable-state. The thermal currents flowing through the junction can be written as $\dot{Q}(\varphi )=\kappa (\varphi ){\rm{\Delta }}T$ as previously mentioned, where *κ*(*ϕ*) is presented in Eq. (17). Considering the interaction between thermal quasiparticles and phonons existing in substrate lattice, heat lossing from *S*_*R*_ into the lattice can be modeled as ${\dot{Q}}_{qp-ph,R}(\varphi )=0.98{\rm{\Sigma }}V({T}_{R}^{5}-{T}_{bath}^{5}){e}^{-{\rm{\Delta }}/({k}_{B}{T}_{R})}$^34–37^, where Σ is the material constant denoting the coupling strength between thermal quasiparticles and phonons^36^, *V* the volume of the superconducting electrode, *T*_*bath*_ the temperature of phonon bath or phonon, and *T*_*R*_ the temperature of *S*_*R*_. Then the temperature *T*_*R*_ can be obtained by the thermal-balance equation^36^

19 $$\dot{Q}(\varphi )-{\dot{Q}}_{qp-ph,R}(\varphi )=0,$$

that is

20 $$\kappa (\varphi ){\rm{\Delta }}T-0.98{\rm{\Sigma }}V({T}_{R}^{5}-{T}_{bath}^{5}){e}^{-{\rm{\Delta }}/({k}_{B}{T}_{R})}=0,$$

where *T*_*bath*_ < *T*_*R*_ < *T*_*L*_.

**Figure 7:**
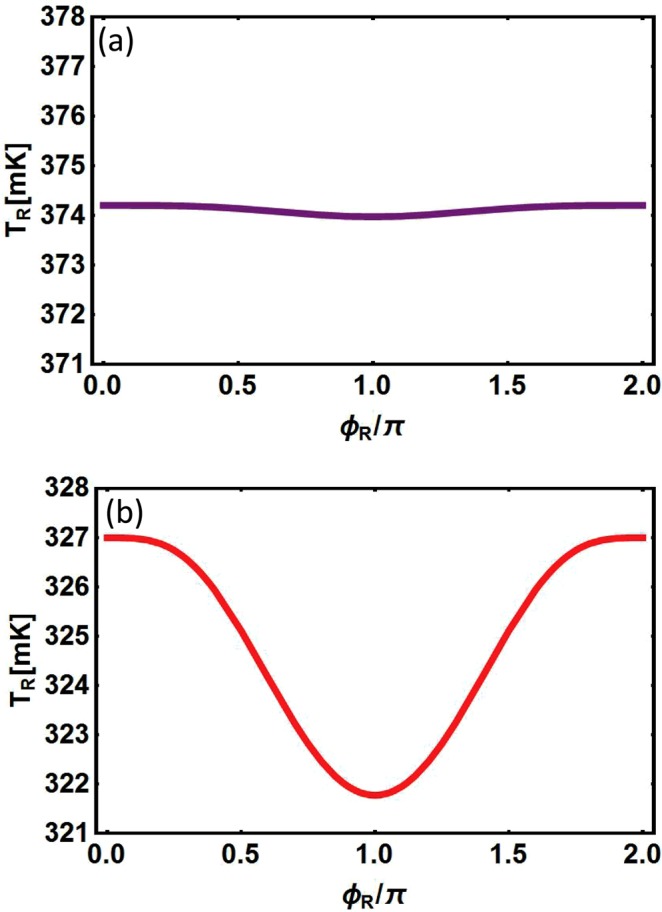
Temperature of the right hand superconducting electrode *T*_*R*_ as a function of *ϕ*_*R*_ for (**a**) *T* = 600 *mK*, (**b**) *T* = 100 *mK*. Here, *μ* = 1.38 *meV*, *h*_0_ = 1.38 *meV*, Δ = 0.15 *meV*, *T*_*L*_ = 450 *mK*, *T*_*bath*_ = 80 *mK* and Σ*V* = 3.6 * 10^−10^ *W*/*K*^5^.

**Figure 8:**
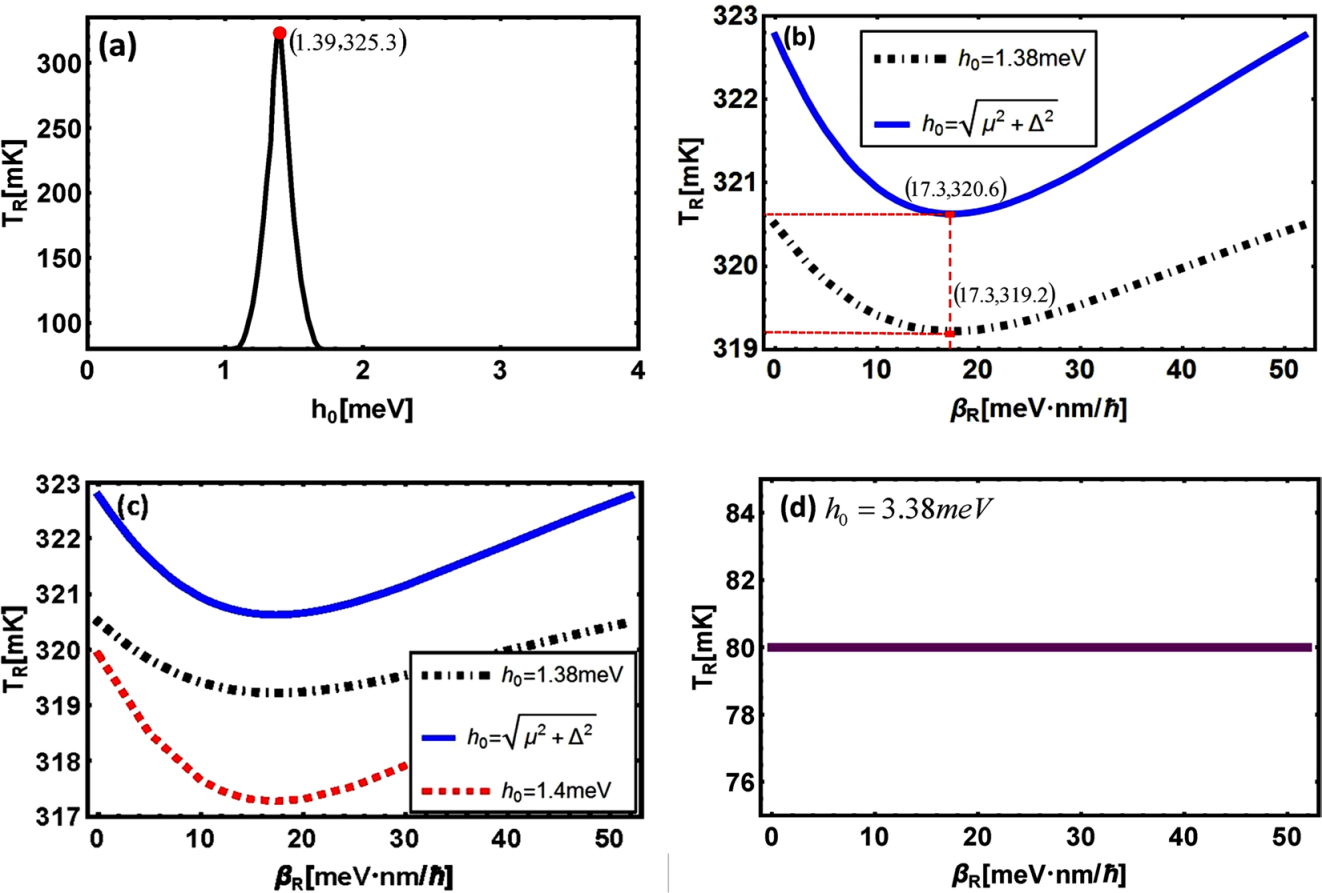
(**a**) *h*_0_-dependence of *T*_*R*_. (**b**–**d**) *β*_*R*_-dependence of *T*_*R*_ with variable *h*_0_. Here, Spin-orbit coupling parameters are the same with that in Fig. 5 and the other parameters are the same with that in Fig. 7.

The resulting behavior of *T*_*R*_ is displayed in Figs 7 and 8. Through comparing Fig. 7(a) with Fig. 7(b) we find that when *h*_0_ takes a fixed value, the changing behavior of *T*_*R*_ as a function of *ϕ*_*R*_ are similar no matter what the temperature *T* takes. It is rather remarkable that the value of *T*_*R*_ at each point of the curve is larger in Fig. 7(a) than in Fig. 7(b), so as the average value of *T*_*R*_, however the change of curve becomes slow. This is because when *T* increases, the amount of thermal excited quasiparticles goes up and the temperature of the right-hand superconductor is raised.

Now we analyze Fig. 8 for the effect of *h*_0_ and *β*_*R*_ on *T*_*R*_ (*λ*_*R*_-dependence of *T*_*R*_ is similar with the situation of *β*_*R*_-dependence so we do not put further consideration). Similar with the situation of thermal conductance, there is an important conclusion that a larger value and a sharper switching behavior of *T*_*R*_ will be obtained when $|\frac{{\lambda }_{R}}{{\beta }_{R}}-\frac{{\lambda }_{L}}{{\beta }_{L}}|$ is larger and the impurity concentration of 2DEG makes *h*_0_ take value at $\sqrt{{\mu }^{2}+{{\rm{\Delta }}}^{2}}$ or around it.

## Conclusions

Through comparison and analysis, we demonstrated that for a short phase-coherent thermal-biased 2DEG Josephson junction, the angle of incidence *θ*_*e*,*h*_ has influence on thermal transport, but normal incidence make the greatest contribution. Moreover, thermal equilibrium temperature *T*, material parameter *h*_0_ and spin-orbit coupling parameters *λ*_*r*_, *β*_*r*_ both have influence on characters of thermal transport. With a fixed *h*_0_, lower *T* leads to a sharper changing behavior while higher *T* increases the value of thermal conductance. On the contrary, increasing *h*_0_ at fixed *T* creates a “peak” for thermal conductance. Through further analysis, we find that a larger value and a sharper changing behavior of thermal conductance will be obtained when the parameter $|\frac{{\lambda }_{R}}{{\beta }_{R}}-\frac{{\lambda }_{L}}{{\beta }_{L}}|$ is larger and the impurity concentration of 2DEG makes *h*_0_ take value as $\sqrt{{\mu }^{2}+{{\rm{\Delta }}}^{2}}$ or around it (For example, ${h}_{0}\in [\sqrt{{\mu }^{2}+{{\rm{\Delta }}}^{2}}-0.01\,meV,\sqrt{{\mu }^{2}+{{\rm{\Delta }}}^{2}}+0.01\,meV]$ in our model), therefore selecting moderately-doped 2DEG materials and superconducting electrodes according to the above method is a valid way to increase thermal currents and obtain a more sensitive Josephson junction. In this paper, only the influence of *λ*_*R*_, *β*_*R*_ is considered, however we infer that *λ*_*L*_, *β*_*L*_ have the same effect on thermal transport as *λ*_*R*_, *β*_*R*_ do according to the symmetry of the junction.

The proposal of a possible experimental setup makes us conscious that with realistic system parameters, this setup consisting of 2DEG Josephson junction can achieve a higher value and a sharper changing behavior of *T*_*R*_ when ${h}_{0}\in [\sqrt{{\mu }^{2}+{{\rm{\Delta }}}^{2}}-0.01\,meV,\sqrt{{\mu }^{2}+{{\rm{\Delta }}}^{2}}+0.01\,meV]$, $|\frac{{\lambda }_{R}}{{\beta }_{R}}-\frac{{\lambda }_{L}}{{\beta }_{L}}|$ is larger. This setup may provide a way to choose suitable 2DEG materials and superconducting electrodes to control the change of temperature and obtain an efficient temperature regulator.

Moreover, through comparison we find that the observations of theoretical model match with the results of experimental setup appropriately, meaning that the experimental method can be realized with the theoretical support.

## Supplementary information

Thermal transport of Josephson junction based on two-dimensional electron gas
