# Supplementary material for: Thermal transport of Josephson junction based on two-dimensional electron gas
Source: Sci Rep. 2019 Feb 18;9:2187. doi: 10.1038/s41598-019-38704-6 (PMC6379384; doi:10.1038/s41598-019-38704-6)
Supplement: Supplementary file 1 — Thermal transport of Josephson junction based on two-dimensional electron gas [file 41598_2019_38704_MOESM1_ESM.pdf]

Thermal transport of Josephson junction based on two-dimensional electron gas

X. X. Luo[1†], Y. F. Peng[1], H. Z. Shen[1,2] & X. X. Yi[1,2*]

[1]*Center for Quantum Sciences, Northeast Normal University, Changchun 130117, China*

[2]*Center for Advanced Optoelectronic Functional Materials Research, and Key Laboratory for UV Light-Emitting Materials and Technology of Ministry of Education, Northeast Normal University, Changchun 130024, China*

† *To whom correspondence should be addressed. E-mail: luoxx231@nenu.edu.cn*

* *To whom correspondence should be addressed. E-mail: yixx050@nenu.edu.cn*

Supplementary Note

Specific expressions of parameters $k_{e_{1r},e_{2r}}$, $k_{h_{1r},h_{2r}}$, $\chi_{e,h}$, $A_{e,h}$, $\vec{k}_{e_{1r},e_{2r}}$, $\vec{k}_{h_{1r},h_{2r}}$, $\vec{r}$ in Eqs.(9) through (12) are written as

$$k_{e_{1r}} = \sqrt{\frac{\mu^2+\omega^2-\Delta^2-h_0^2+2\sqrt{\Delta^2 h_0^2-\Delta^2\mu^2+\mu^2\omega^2}}{\hbar^2\left(\lambda_r^2+\beta_r^2-2\lambda_r\beta_r\sin 2\theta_{ei}\right)}},$$

$$k_{e_{2r}} = \sqrt{\frac{\mu^2+\omega^2-\Delta^2-h_0^2+2\sqrt{\Delta^2 h_0^2-\Delta^2\mu^2+\mu^2\omega^2}}{\hbar^2\left(\lambda_r^2+\beta_r^2+2\lambda_r\beta_r\sin 2\theta_{ei}\right)}},$$

$$k_{h_{1r}} = \sqrt{\frac{\mu^2+\omega^2-\Delta^2-h_0^2-2\sqrt{\Delta^2 h_0^2-\Delta^2\mu^2+\mu^2\omega^2}}{\hbar^2\left(\lambda_r^2+\beta_r^2-2\lambda_r\beta_r\sin 2\theta_{hi}\right)}},$$

$$k_{h_{2r}} = \sqrt{\frac{\mu^2+\omega^2-\Delta^2-h_0^2-2\sqrt{\Delta^2 h_0^2-\Delta^2\mu^2+\mu^2\omega^2}}{\hbar^2\left(\lambda_r^2+\beta_r^2+2\lambda_r\beta_r\sin 2\theta_{hi}\right)}},$$

$$\chi_e = (\mu+\omega)^2 - h_0^2 - \Delta^2 - \hbar^2 k_{e_{1r}}^2\left(\lambda_r^2+\beta_r^2-2\lambda_r\beta_r\sin 2\theta_{ei}\right),$$

$$\chi_h = (\mu+\omega)^2 - h_0^2 - \Delta^2 - \hbar^2 k_{h_{1r}}^2 \left(\lambda_r^2 + \beta_r^2 - 2\lambda_r\beta_r \sin 2\theta_{hi}\right),$$

$$A_e = 4\Delta^2(\mu+h_0)^2\hbar^2 k_{e_{1r}}^2 \left(\lambda_r^2+\beta_r^2-2\lambda_r\beta_r\sin 2\theta_{ei}\right) + \Delta^2[2\left(\mu+h_0\right)\left(-\mu+h_0-\omega\right)+\chi_e]^2$$
$$+ \left[2\Delta^2\left(\mu+h_0\right)+\left(\mu+h_0-\omega\right)\chi_e\right]^2 + \chi_e^2\hbar^2 k_{e_{1r}}^2\left(\lambda_r^2+\beta_r^2-2\lambda_r\beta_r\sin 2\theta_{ei}\right),$$

$$A_h = 4\Delta^2(\mu+h_0)^2\hbar^2 k_{h_{1r}}^2 \left(\lambda_r^2+\beta_r^2-2\lambda_r\beta_r\sin 2\theta_{hi}\right) + \Delta^2[2\left(\mu+h_0\right)\left(-\mu+h_0-\omega\right)+\chi_h]^2$$
$$+ \left[2\Delta^2\left(\mu+h_0\right)+\left(\mu+h_0-\omega\right)\chi_h\right]^2 + \chi_h^2\hbar^2 k_{h_{1r}}^2\left(\lambda_r^2+\beta_r^2-2\lambda_r\beta_r\sin 2\theta_{hi}\right),$$

$$\vec{k}_{e_{1r}} = k_{e_{1r}}\left(\cos\theta_{ei}, \sin\theta_{ei}\right),$$

$$\vec{k}_{e_{2r}} = k_{e_{2r}}\left(\cos\left(\pi-\theta_{ei}\right), \sin\left(\pi-\theta_{ei}\right)\right),$$

$$\vec{k}_{h_{1r}} = k_{h_{1r}}\left(\cos\theta_{hi}, \sin\theta_{hi}\right),$$

$$\vec{k}_{h_{2r}} = k_{h_{2r}}\left(\cos\left(\pi-\theta_{hi}\right), \sin\left(\pi-\theta_{hi}\right)\right),$$

$$\vec{r} = (x, y).$$

Supplementary Equations

$$t_e = -\frac{\left[\begin{array}{l} \left(-a_4b_3c_2+a_3b_4c_2+a_4b_2c_3-a_2b_4c_3-a_3b_2c_4+a_2b_3c_4\right)d_{1L} \\ +\left(a_4b_3c_{1L}-a_3b_4c_{1L}-a_4b_{1L}c_3+a_{1L}b_4c_3+a_3b_{1L}c_4-a_{1L}b_3c_4\right)d_2 \\ +\left(-a_4b_2c_{1L}+a_2b_4c_{1L}+a_4b_{1L}c_2-a_{1L}b_4c_2-a_2b_{1L}c_4+a_{1L}b_2c_4\right)d_3 \\ +\left(a_3b_2c_{1L}-a_2b_3c_{1L}-a_3b_{1L}c_2+a_{1L}b_3c_2+a_2b_{1L}c_3-a_{1L}b_2c_3\right)d_4 \end{array}\right]}{\left[\begin{array}{l} \left(a_4b_3c_2-a_3b_4c_2-a_4b_2c_3+a_2b_4c_3+a_3b_2c_4-a_2b_3c_4\right)d_{1R} \\ +\left(-a_4b_3c_{1R}+a_3b_4c_{1R}+a_4b_{1R}c_3-a_{1R}b_4c_3-a_3b_{1R}c_4+a_{1R}b_3c_4\right)d_2 \\ +\left(a_4b_2c_{1R}-a_2b_4c_{1R}-a_4b_{1R}c_2+a_{1R}b_4c_2+a_2b_{1R}c_4-a_{1R}b_2c_4\right)d_3 \\ +\left(-a_3b_2c_{1R}+a_2b_3c_{1R}+a_3b_{1R}c_2-a_{1R}b_3c_2-a_2b_{1R}c_3+a_{1R}b_2c_3\right)d_4 \end{array}\right]}\frac{e^{ik_{e_{1L}}y\sin\theta_{ei}}}{e^{ik_{e_{1R}}y\sin\theta_{ei}}} \tag{S1}$$

$$t_h = \frac{\left[\begin{array}{l} \left(a_3 b_2 c_{1R} - a_2 b_3 c_{1R} - a_3 b_{1R} c_2 + a_{1R} b_3 c_2 + a_2 b_{1R} c_3 - a_{1R} b_2 c_3\right) d_{1L} \\ + \left(-a_3 b_2 c_{1L} + a_2 b_3 c_{1L} + a_3 b_{1L} c_2 - a_{1L} b_3 c_2 - a_2 b_{1L} c_3 + a_{1L} b_2 c_3\right) d_{1R} \\ + \left(a_3 b_{1R} c_{1L} - a_{1R} b_3 c_{1L} - a_3 b_{1L} c_{1R} + a_{1L} b_3 c_{1R} + a_{1R} b_{1L} c_3 - a_{1L} b_{1R} c_3\right) d_2 \\ + \left(-a_2 b_{1R} c_{1L} + a_{1R} b_2 c_{1L} + a_2 b_{1L} c_{1R} - a_{1L} b_2 c_{1R} - a_{1R} b_{1L} c_2 + a_{1L} b_{1R} c_2\right) d_3 \end{array}\right]}{\left[\begin{array}{l} \left(-a_4 b_3 c_2 + a_3 b_4 c_2 + a_4 b_2 c_3 - a_2 b_4 c_3 - a_3 b_2 c_4 + a_2 b_3 c_4\right) d_{1R} \\ + \left(a_4 b_3 c_{1R} - a_3 b_4 c_{1R} - a_4 b_{1R} c_3 + a_{1R} b_4 c_3 + a_3 b_{1R} c_4 - a_{1R} b_3 c_4\right) d_2 \\ + \left(-a_4 b_2 c_{1R} + a_2 b_4 c_{1R} + a_4 b_{1R} c_2 - a_{1R} b_4 c_2 - a_2 b_{1R} c_4 + a_{1R} b_2 c_4\right) d_3 \\ + \left(a_3 b_2 c_{1R} - a_2 b_3 c_{1R} - a_3 b_{1R} c_2 + a_{1R} b_3 c_2 + a_2 b_{1R} c_3 - a_{1R} b_2 c_3\right) d_4 \end{array}\right]} \frac{e^{ik_{e_{1L}} y \sin\theta_{ei}}}{e^{ik_{h_{2R}} y \sin\theta_{hi}}} \tag{S2}$$
